# Pneumothorax Secondary to a Traumatic Brazilian Jiu-Jitsu Injury

**DOI:** 10.7759/cureus.54941

**Published:** 2024-02-26

**Authors:** Satnam Singh, Harpreet Singh, Birkaran Sadhar, Smaran S Teru

**Affiliations:** 1 Trauma, Lake Erie College of Osteopathic Medicine, Erie, USA; 2 Internal Medicine, Lake Erie College of Osteopathic Medicine, Erie, USA; 3 Ophthalmology, Lake Erie College of Osteopathic Medicine, Erie, USA; 4 Family Medicine, Lake Erie College of Osteopathic Medicine, Erie, USA

**Keywords:** jiu-jitsu, thoracic injuries, sports, pneumothorax, trauma

## Abstract

A pneumothorax is a medical condition characterized by the presence of free air in the pleural cavity. Pneumothorax can be classified as spontaneous, traumatic, or iatrogenic. Spontaneous pneumothorax sustained from a jiu-jitsu-induced blunt trauma has not been described in any sports literature. This case report involves a 26-year-old male athlete who presented to the emergency room complaining of right-sided chest pain in the recumbent position and shortness of breath upon exertion. Breath sounds were diminished on the right with hyper resonance to percussion. Inspection of the chest revealed diffuse erythema on the right side. A chest X-ray revealed a right tension pneumothorax that was treated with a 20-French chest tube. This report aims to highlight the importance of recognizing the possibility of pneumothorax in jiu-jitsu athletes, implementing early treatment, and exploring potential causes of pneumothorax in otherwise healthy individuals.

## Introduction

Brazilian jiu-jitsu (BJJ) is a grappling-centric combat sport designed to assert control over a resisting adversary, ultimately culminating in a submission. BJJ is a rapidly growing sport in America. The total number of people practicing BJJ has increased by 23% between 2010 and 2021 [1]. The primary objective involves employing an array of submissions to secure a victory over the opponent. The submissions range from joint locks, where the joint is extended beyond the anatomical limit, to chokeholds that reduce the blood supply to the brain. The match starts with both participants standing in opposition, aiming to take each other to the ground to secure positional control and submit the opposing party [2]. Given the physical nature of the sport, injuries are to be expected.

The most common self-identified injuries encountered during BJJ practice manifest in the hand/fingers, followed by the arm/elbows, foot/toes, and subsequently the back, in descending order of occurrence [3]. In a study by Hunker et al., conducted at 234 BJJ schools across the country, the most common injuries were to the finger/hand (78.6% of participants), wrist/forearm (28.2% of participants), elbow/upper arm (30.8% of participants), shoulder (48.7% of participants), head/face (23.1% of participants), and the neck (28.2% of participants) [4]. The data was collected through surveys filled out by the individual athletes at their BJJ schools in which they self-reported the injuries they had experienced. Unfortunately, the existing literature lacks comprehensive data on injuries associated with BJJ and, within the available data, there is not a single documented case of a pneumothorax sustained from a BJJ injury.

Pneumothorax, defined as the presence of air within the pleural cavity, can manifest spontaneously or traumatically. Spontaneous occurrences are often linked to the rupture of subpleural blebs or bullae [5]. When associated with an underlying lung ailment such as pneumonia, asthma, or cystic fibrosis, it is termed secondary spontaneous pneumothorax; otherwise, it is referred to as primary spontaneous pneumothorax [6]. Traumatic pneumothorax, on the other hand, can be caused by penetrating or blunt trauma, with the latter causing lung injury through direct impact or rib fracture-induced laceration [6].

While not commonly observed in sports, pneumothorax following blunt chest trauma has been documented in various athletic activities, including ice hockey, snowboarding, skiing, cycling, and football [7]. BJJ, however, has never been reported in such documented cases. This case report delves into an instance of a pneumothorax resulting from blunt trauma while practicing BJJ, shedding light on a unique occurrence within the realm of sports-related injuries.

## Case presentation

A 26-year-old, non-smoking male presented to the emergency department complaining of right-sided chest pain and shortness of breath approximately 48 hours following a chest impact sustained while practicing BJJ. The patient was taken down to the floor by his opponent from a throw. Upon landing on the floor, the patient received an impact on his chest from the weight of his opponent landing on his chest. The patient complained of pain on the right side of his chest immediately post-impact and felt shortness of breath 10 minutes later. Before presenting to the ED, the patient noted worsening pleuritic chest pain and dyspnea in the supine position. His discomfort was relieved by maintaining an erect posture, requiring him to sleep upright in a chair.

**Figure 1:**
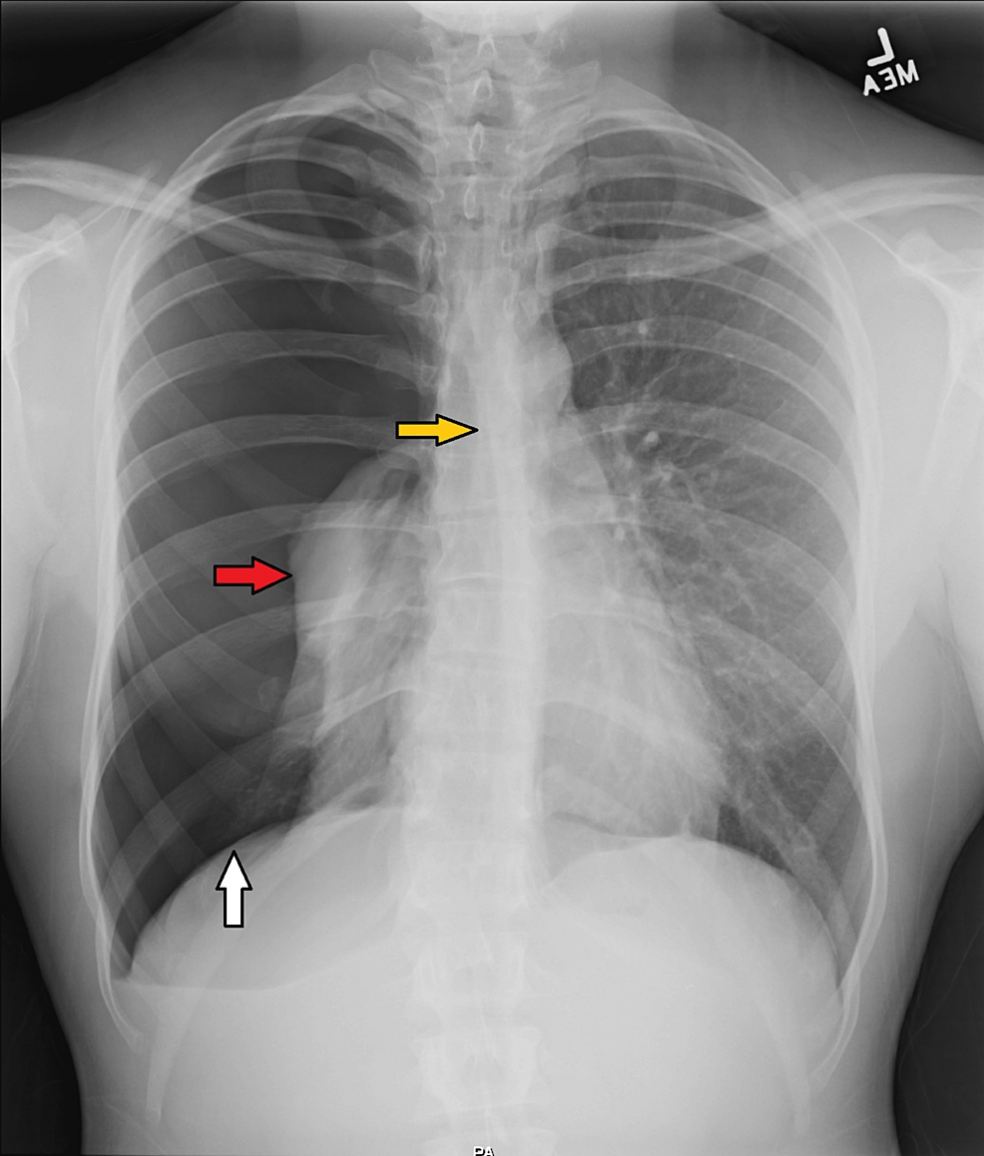
Chest X-ray showing complete right pneumothorax (red arrow). Developing mild mediastinal shift is noted (yellow arrow) with depression of the right hemidiaphragm suggesting tension (white arrow).

Emergency room examination revealed a right-sided pneumothorax. the patient’s vital signs included a temperature of 98.1^o^F, pulse of 105 beats per minute, respiratory rate of 16 breaths per minute, blood oxygen saturation of 93%, and a blood pressure of 122/65 mmHg. The patient rated the pain on presentation as a constant 7/10 since the trauma was experienced. The patient denied nausea, vomiting, hematemesis, and cough. The patient did not have any significant medical history but did fall under the category of the tall and slim build that is prone to pneumothoraces. However, physical exam findings of 182.88 cm for height and 68.04 kg for weight were not concerning for marfanoid habitus or Ehlers-Danlos syndrome. The patient reported not using cigarettes, vaping devices, or marijuana. Following the patient’s history, chest radiography was performed, which revealed a large complete right pneumothorax with developing mild mediastinal shift to the left and depression of the right hemidiaphragm suggesting tension (Figure 1). The patient did not meet the clinical criteria for a tension pneumothorax on physical exam. The heart was normal in size and the osseous structures were intact. Electrocardiography was obtained as well and was unremarkable.

**Figure 2:**
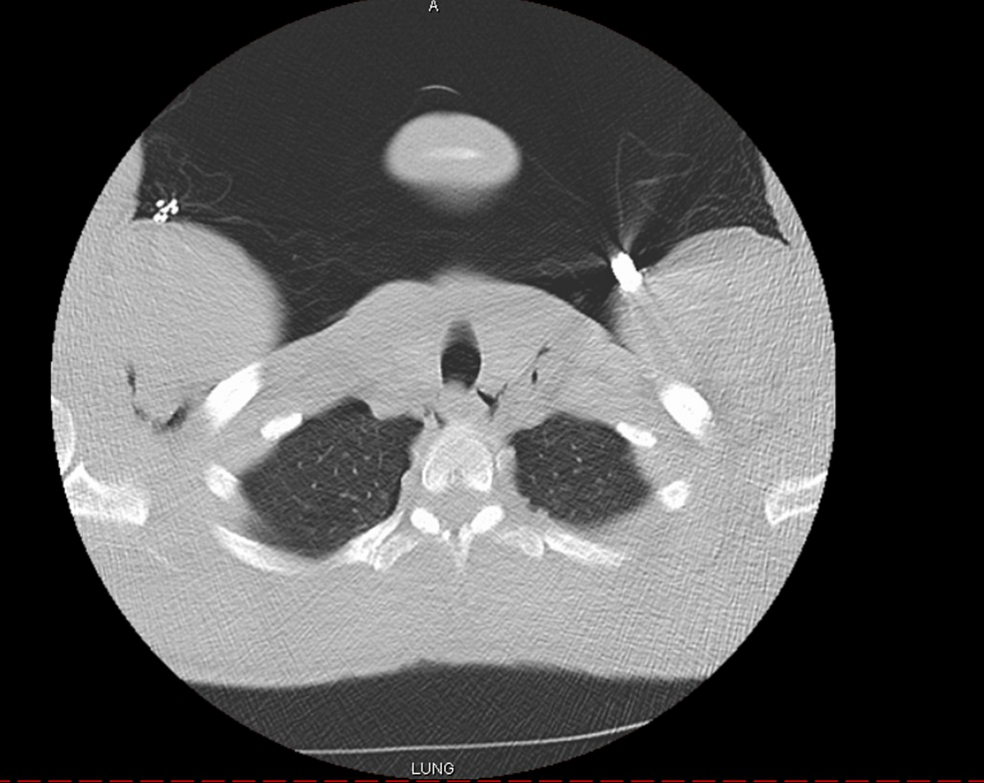
Axial computed tomography slice of the apical lungs highlighting the absence of pulmonary blebs.

Given the patient’s history of a traumatic chest injury and his clinical presentation, he was admitted for further evaluation. The general surgery team was consulted to place a 20-French right thoracostomy tube in the fourth intercostal space at the mid-axillary line. The chest tube was placed on a Pleur-evac and on suction. The chest x-ray done post placement of the chest tube revealed full expansion of the lung. The patient was placed in the ICU for the following two days. In the ICU, the patient rated the pain as a 7/10. On the first day in the ICU, the patient requested and received Dilaudid 1 mg IVP, which brought the pain down to a 4/10. On the second day, the patient received Toradol 30 mg IVP in the morning and Dilaudid 1 mg IVP in the afternoon for pain management. Throughout the remainder of the patient’s hospital stay, he was given Tylenol 650 mg orally every six hours/PRN (pro re nata). Pulmonary blebs are typically found in the lung apices. On day two of admission, the patient’s CT chest without contrast showed a clear left lung without any blebs, and no definite rib fracture (Figure 2). 

**Figure 3:**
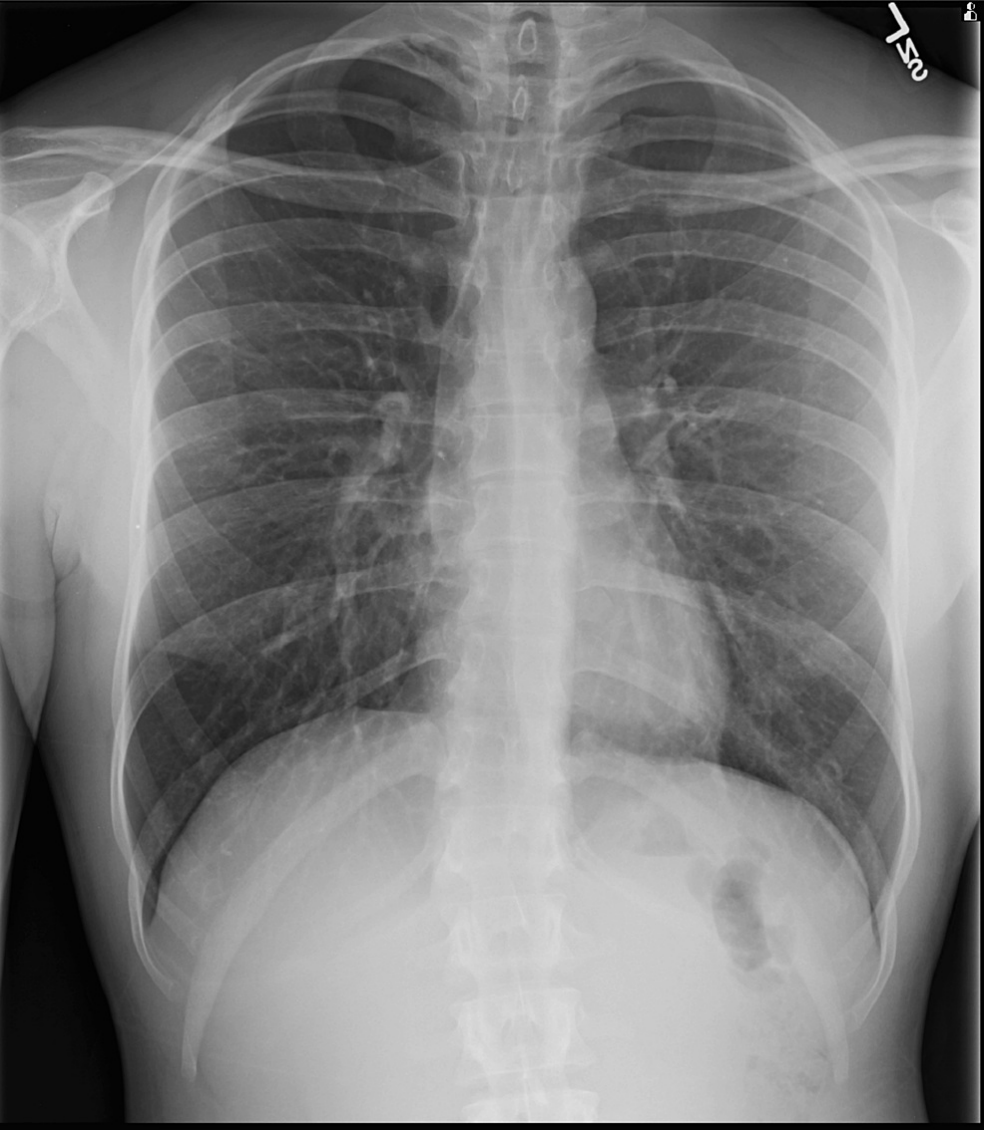
Chest X-ray showing resolution of pneumothorax.

Repeat chest x-rays were performed daily. The chest tube was removed on day 11 (January 24), and the patient was discharged shortly after with an 8 mm apical right pneumothorax. Chest x-ray post-treatment was done on January 28th (Figure 3), which showed complete resorption of the apical pneumothorax. The patient had a follow-up appointment again after seven days, in which he was advised to take a six-week break from sports.

## Discussion

The presented case outlines a unique instance of traumatic pneumothorax resulting from BJJ-induced blunt chest trauma. Chest trauma sustained during sporting activities is extremely rare; the American Journal of Sports Medicine reviewed over 1.5 million high school athlete injuries and found an incidence of 7.4-28 cases of pneumothorax per 100,000 athlete exposures (which includes practices and games) [8]. A BJJ-induced pneumothorax has not been described in medical literature before.

The pneumothorax manifested in a typical manner in the patient. The patient experienced the common symptoms of respiratory discomfort, hyperresonant percussion notes, and absent breath sounds [6]. The patient waited 48 hours to seek treatment since he assumed the chest pain was possibly due to a chest contusion secondary to blunt trauma. Chest pain is a common symptom of a chest contusion [9]. However, the absence of breath sounds over the affected lung would signal the more likely diagnosis of a pneumothorax [10]. Delays in treatment can be fatal due to the formation of a tension pneumothorax. A tension pneumothorax can cause a mediastinal shift which is a medical emergency because trapped air in the pleural space elevates chest pressure leading to compression of major vessels and compromising cardiac output. The opposite lung may also become compressed, resulting in profound respiratory distress leading to shock and subsequent death [11].

Vaping and smoking are associated with increased risk of pneumothorax [12,13], but this patient did not engage in any of these behaviors. Determining the etiology of a pneumothorax can help guide treatment. First, it should be established whether the pneumothorax is spontaneous or traumatic. If spontaneous, then the provider should ascertain whether it is a primary or secondary pneumothorax. Secondary pneumothorax can be caused by a variety of conditions such as tuberculosis, necrotizing pneumonia, pneumocystis carini, lung cancer, sarcoma involving the lung, sarcoidosis, endometriosis, cystic fibrosis, acute severe asthma, idiopathic pulmonary fibrosis, etc [14]. If caused by one of these secondary conditions, treatment of the underlying condition should be addressed to prevent recurrence [15].

As there was an absence of other secondary conditions, smoking/vaping habits, or fractured ribs that might have caused a lung to rupture, the pneumothorax was thought to potentially result from the rupture of a pulmonary bleb. A pulmonary bleb refers to a small accumulation of air situated between the lung and the outer layer of the lung (visceral pleura), commonly located in the upper lobe of the lung. If a bleb ruptures, then the released air enters the chest cavity, leading to a pneumothorax, characterized by the presence of air between the lung and the chest cavity [15]. The mechanism for the bleb rupture could have been due to the “paper bag effect”, which occurs when someone inhales deeply, similar to holding their breath before an abrupt impact, while concurrently sealing the airway by closing the epiglottis. The simultaneous action creates elevated pressure in the lungs, and with a closed airway, it can result in the escape of air from the lung into the pleural space between the lung and chest wall, ultimately causing a pneumothorax [16].

The patient was not given an exact time for return to play but was recommended to wait at least six weeks until returning to playing sports. As of now, there is no consensus on the time frame for return to play post pneumothorax. The literature often uses ranges between 2-10 weeks [17]. This may be an area for further exploration, establishing guidelines to help physicians determine what factors should be used when giving patients a timeline for return to play.

This case underscores the importance of recognizing pneumothorax in athletes, even with mild symptoms, especially in sports where blunt chest trauma is inherent. The tall and slim physique of this patient may have contributed to the unique presentation of tension pneumothorax in this population. The successful management and return to normal activities highlight the significance of prompt diagnosis and intervention. Healthcare providers and team physicians should be vigilant, considering pneumothorax as a potential consequence of blunt chest trauma in athletes. 

## Conclusions

This case underscores the importance of recognizing a pneumothorax in athletes, even with mild symptoms, especially in sports where blunt chest trauma is inherent. This case adds valuable insights to the limited literature on trauma-induced pneumothorax in the setting of BJJ and emphasizes the need for continued awareness and research in the field of sports-related thoracic injuries. The successful management and return to normal activities highlight the significance of prompt diagnosis and intervention. Healthcare providers and team physicians should be vigilant, considering pneumothorax as a potential consequence of blunt chest trauma in athletes. 
